# Digital Health Navigation: An Enabling Infrastructure for Optimizing and Integrating Virtual Care Into Oncology Practice

**DOI:** 10.1200/CCI.21.00111

**Published:** 2021-11-29

**Authors:** Anaeze C. Offodile, Allison J. Seitz, Susan K. Peterson

**Affiliations:** ^1^ Department of Plastic Surgery, UT MD Anderson Cancer Center, Houston, TX; ^2^ Department of Health Services Research, UT MD Anderson Cancer Center, Houston, TX; ^3^ Baker Institute for Public Policy, Rice University, Houston, TX; ^4^ McGovern Medical School, UT Health Science Center, Houston, TX; ^5^ Department of Behavioral Science, UT MD Anderson Cancer Center, Houston, TX

## Introduction

As technological advances over the past decade have led to the miniaturization and affordability of consumer-facing wearables and digital wellness applications, these tools have taken on an increasing role in care delivery. The social distancing and self-quarantine requirements of the COVID-19 pandemic have significantly accelerated this trend.^1^ For example, consumer wearables, as well as other remote monitoring devices, have been increasingly implemented over the past year to remotely detect COVID-19 infections through continual physiologic data and temperature monitoring.^2,3^ However, it is imperative to ensure the quality of these aforementioned devices and applications, as inaccuracies can negatively affect clinical decision making and patient outcomes.^4^

The impetus is that these devices will augment traditional health care services by enhancing patient access while simultaneously engendering self-care and patient activation. However, there remains considerable room for improvement with respect to the provision of virtual care services within the context of oncology. Specifically, there is a need for thoughtful implementation of the clinical workflows supporting these technology solutions, more robust frontline provider education, and consideration around primary access barriers for marginalized communities. The latter is salient because vulnerable populations (ie, low socioeconomic status, racial minorities, and rural settings) are characterized by poor broadband access, limited English proficiency, low income, and minimal health or technological literacy.^5,6^ Furthermore, the existing evidence base for the effectiveness of digital health interventions (DHIs), such as telehealth, remote patient monitoring, and digital phenotyping, has been limited and yet to be scaled in oncology clinical practice.^7-9^ We posit that the development of a new provider role within the oncology ecosystem, termed a digital health navigator (DHN), will help address many of the aforementioned concerns and harness the full potential of virtual care in oncology. Such a role has been previously described for behavioral health services in psychiatric patients^10^; however, to the best of our knowledge, it has not been contextualized to oncology. Through the implementation of this role, we believe that digital inclusion for all patients with cancer is attainable, regardless of their background or knowledge level. This role will also help drive a digital therapeutic alliance between patients and the oncology care team that improves outcomes, patient experience, and reduces health care costs.

## Job Description

The utility of DHN was first identified by Ben-Zeev and Drake in a 2015 article, wherein they outlined such patient-facing functions as providing education on the basic operation of devices, delivering culturally competent feedback on the data abstracted from these devices (ie, vital signs, patient reported outcomes, and sleep patterns), and identifying any follow-up action.^11^ The intent, limitations, strengths, supporting evidence, and associated costs of these technologies will also be provided.^11^ DHNs can help assess patient preferences, interest, literacy, and dexterity around these tools.^11^ Of note, patients with cancer are a unique population who may face additional obstacles such as fragmented care, complicated treatment regimens, and inadequate communication with providers.^12-15^ For example, Kitamura et al^16^ found that many patients reported anxiety regarding the use of new technology, as well as a reluctance or difficulty to communicate with their providers, when using telehealth services for oncologic consultations.

For providers, DHNs can leverage up-to-date practical and regulatory knowledge to support and train the oncology care team. They will help streamline clinical workflows, troubleshoot implementation bottlenecks, personalize user interfaces, facilitate technological navigation (ie, patient portal access and utilization), and disseminate best practice, all with an eye toward maximizing the productivity associated with virtual care paradigms. Finally, it is highly plausible that a spillover effect of DHN utilization may improve provider well-being, and this is because of their ability to offset many nonpatient care elements of the clinical team's workload, often cited as a point of frustration, anxiety, and dissatisfaction.^17,18^

DHNs should ideally be embedded within a comprehensive cancer care team, and this alignment best enables them to coordinate the provision of DHIs, address barriers to timely receipt of virtual care throughout all phases of the cancer care continuum, and gather data on trends in DHI utilization. DHNs can serve as a critical link between patients and providers, able to right-size DHIs for both stakeholder groups. Qualifications should be flexible, site-specific, and underpinned by a dynamic curriculum of continuous education on emerging technology platforms and digital tools (Table 1). Consistent with the scope of the role, antecedent professional backgrounds can be broad including but not limited to clinicians, nursing, case management, office staff, and medical assistants.^1^ However, a history of working collaboratively with clinicians, administrators, and patients is essential along with emotional intelligence, experience with electronic medical records, and familiarity with medical terminology and standard clinical practices. Other core competencies should be informed by the skills necessary to drive DHI adoption, within a particular use case, by patients and clinicians as a one-size-fits-all approach is impractical (Table 1).

**TABLE 1.**
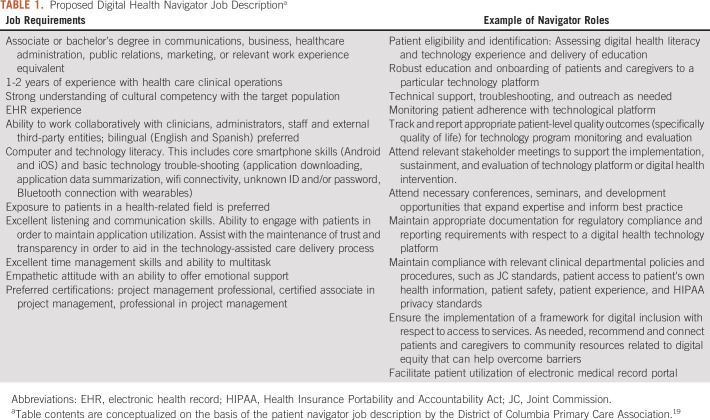
Proposed Digital Health Navigator Job Description^a^

## Metrics of Success

The magnitude of success in the coordination of DHIs through DHNs can be assessed through a variety of patient-related outcomes, ideally tailored to the specific use case. Examples include patient retention, improvements in health-related quality of life, patient engagement and self-care, and reductions in acute care visits and treatment disruptions. These measures will need to be examined in future studies to promote widespread utilization of DHNs. For example, future researchers may prospectively assess the impact of the implementation of the DHN role on patient-reported outcomes such as EuroQol-5 Dimensions, Patient Activation Measure, Functional Assessment of Cancer Therapy-General, and MD Anderson Symptom Inventory. Additionally, associated variation in the rates of acute care utilization (emergency room visits and hospitalizations) and total health care spending on an episode-of-care basis (30- or 90-day episode) can also be assessed in a randomized or matched case-control basis. In addition to remote patient monitoring and technical support, DHNs will also be responsible for tracking patient adherence to clinician recommendations around DHI use. Finally, it will be crucial to also evaluate the long-term financial impacts associated with the DHN role, as it is highly unlikely to be directly reimbursed. The workload and number of patients taken on by each DHN will be greatly dependent on the health care setting (ie, clinic *v* hospital) and practice-based needs, and thus, it will need to be assessed on a case-by-case basis as this will factor into long-term financial costs. A useful benchmark to consider might be the published caseload for a layperson patient navigator, which is approximately 152 patients per quarter.^20^ Approaches such as time-driven activity-based costing and cost-effectiveness analysis via decision trees and Markov simulation models will be beneficial in articulating a sustainable value proposition for DHNs to hospital or clinical practice administration.

## Path Forward

To ensure successful execution of the DHN position within the health care system, staffing models and scope of practice must be clearly defined. Similarly, the training process needs to be standardized. Wisniewski et al^1^ have previously proposed a 10-hour training and certification process that may be used as a framework in enacting this new role in an oncologic context. Much like traditional patient navigators who address barriers to clinical access and serve to reduce health disparities in marginalized communities, DHNs can similarly play an integral role in improving access and quality of care. As health systems continue to rapidly evolve with respect to the incorporation of digital health services within patient care, DHNs are uniquely positioned to promote health equity and patient-centered care. Given the tremendous potential for DHNs in oncology, it will be imperative to apply both change management methodologies and implementation science principles to better guide the durable deployment of this new health care role.

In conclusion, DHIs can provide a cost-effective, efficient, and safer way to remotely monitor and meaningfully engage with patients with cancer. Prior research has demonstrated that telehealth interventions may be beneficial within oncologic patients as these interventions can help reduce treatment burden and minimize disruption to these patients' lives.^21^ Therefore, DHNs are an avenue toward realizing and maximizing the benefits of DHIs in oncology. The thoughtful implementation of digital health navigation will allow *all* patients with cancer to navigate the ongoing digital transformation of oncology care successfully and confidently.

## References

[1] Wisniewski H, Gorrindo T, Rauseo-Ricupero N, et al: The role of digital navigators in promoting clinical care and technology integration into practice. Digit Biomark 4:119-135, 2020 33442585 10.1159/000510144 PMC7768140

[2] Smarr BL, Aschbacher K, Fisher SM, et al: Feasibility of continuous fever monitoring using wearable devices. Sci Rep 10:1-11, 2020 33318528 10.1038/s41598-020-78355-6 PMC7736301

[3] Vindrola-Padros C, Sidhu MS, Georghiou T, et al: The implementation of remote home monitoring models during the COVID-19 pandemic in England. EClinicalMedicine 34:100799, 2021 33817610 10.1016/j.eclinm.2021.100799 PMC8008987

[4] Bent B, Goldstein BA, Kibbe WA, et al: Investigating sources of inaccuracy in wearable optical heart rate sensors. NPJ Digit Med 3:18, 2020 32047863 10.1038/s41746-020-0226-6 PMC7010823

[5] Weinstein JN, Geller A, Negussie Y, et al: Communities in Action: Pathways to Health Equity. Washington, DC, National Academies Press, 2017 28418632

[6] Nouri S, Khoong EC, Lyles CR, et al: Addressing equity in telemedicine for chronic disease management during the Covid-19 pandemic. NEJM Catal Innov Care Deliv 10.1056/CAT.20.0123

[7] Murray E, Hekler EB, Andersson G, et al: Evaluating digital health interventions: Key questions and approaches. Am J Prev Med 51:843-851, 2016 27745684 10.1016/j.amepre.2016.06.008 PMC5324832

[8] Gordon WJ, Landman A, Zhang H, et al: Beyond validation: Getting health apps into clinical practice. NPJ Digit Med 3:14, 2020 32047860 10.1038/s41746-019-0212-z PMC6997363

[9] Leigh S, Ashall-Payne L: The role of health-care providers in mHealth adoption. Lancet Digit Heal 1:e58-e59, 2019 10.1016/S2589-7500(19)30025-1 33323231

[10] Wisniewski H, Torous J: Digital navigators to implement smartphone and digital tools in care. Acta Psychiatr Scand 141:350-355, 2020 31930477 10.1111/acps.13149 PMC7928068

[11] Ben-Zeev D, Drake R, Marsch L: Clinical technology specialists: Who needs them? We do. BMJ 350:h945, 2015 25697164 10.1136/bmj.h945

[12] Galligioni E, Piras EM, Galvagni M, et al: Integrating mHealth in oncology: Experience in the province of trento. J Med Internet Res 17:e114, 2015 25972226 10.2196/jmir.3743 PMC4468599

[13] Pedersen JK, Engholm G, Skytthe A, et al: Cancer and aging: Epidemiology and methodological challenges. Acta Oncol 55:7-12, 2016 (suppl 1) 26825001 10.3109/0284186X.2015.1114670 PMC4957549

[14] Ritchie CS, Kvale E, Fisch MJ: Multimorbidity: An issue of growing importance for oncologists. JCO Oncol Pract 7:371-374, 2011 10.1200/JOP.2011.000460 PMC3219463 22379419

[15] Gilligan T, Coyle N, Frankel RM, et al: Patient-clinician communication: American Society of Clinical Oncology consensus guideline. J Clin Oncol 35:3618-3632, 2017 28892432 10.1200/JCO.2017.75.2311

[16] Kitamura C, Zurawel-Balaura L, Wong RKS: How effective is video consultation in clinical oncology? A systematic review. Curr Oncol 17:17-27, 2010 10.3747/co.v17i3.513 PMC2880899 20567623

[17] Kline RM, Rocque GB, Rohan EA, et al: Patient navigation in cancer: The business case to support clinical needs. JCO Oncol Pract 15:585-590, 2019 10.1200/JOP.19.00230 PMC8790714 31509483

[18] Dyrbye LN, Shanafelt TD, Sinsky CA, et al: Burnout among health care professionals: A call to explore and address this underrecognized threat to safe, high-quality care. NAM Perspect 7, 2017. 10.31478/201707b

[19] http://dcpca.org/includes/storage/brio/files/219/DCPCA%20Cancer%20Patient%20Navigator%20Job%20Description%202019%20FINAL.pdf

[20] Rocque GB, Partridge EE, Pisu M, et al: The patient care connect program: Transforming health care through lay navigation. JCO Oncol Pract 12:e633-e642, 2016 10.1200/JOP.2015.008896 PMC5702802 27165489

[21] Cox A, Lucas G, Marcu A, et al: Cancer survivors’ experience with telehealth: A systematic review and thematic synthesis. J Med Internet Res 19:e11, 2017 28069561 10.2196/jmir.6575 PMC5259589

